# Diagnosis and Treatment of Cerebral Venous Thrombosis: A Review

**DOI:** 10.3389/fnagi.2018.00002

**Published:** 2018-01-30

**Authors:** Yaxi Luo, Xin Tian, Xuefeng Wang

**Affiliations:** ^1^Chongqing Key Laboratory of Neurology, Department of Neurology, The First Affiliated Hospital of Chongqing Medical University, Chongqing, China; ^2^Center of Epilepsy, Beijing Institute for Brain Disorders, Beijing, China

**Keywords:** cerebral venous thrombosis, clinical manifestation, diagnosis, treatment, prognosis

## Abstract

Cerebral venous thrombosis (CVT), also called cerebral venous sinus thrombosis (CVST), is a cerebrovascular disease with diverse clinical manifestations that often affects young adults, women of childbearing age, and children. It's most common clinical manifestations are headache, seizures, altered consciousness, and neurological focal signs on physical examination. CVT can manifest as a single symptom, or it can present as a syndrome consisting of multiple symptoms. This non-specific clinical picture makes diagnosing CVT difficult. Although the mortality rate of CVT has been significantly reduced by improvements in treatment and diagnostic techniques, the mortality rate of severe CVT remains as high as 34.2%. Survivors of this type of CVT have varying degrees of residual symptoms and are not able to return to their previous work. Hence, we performed a comprehensive literature search in the PubMed, EMBASE, and Medline databases to review the diagnosis and treatment of CVT.

CVT is a special type of cerebrovascular disease that present with focal cerebral edema, venous cerebral infarction, seizures, and intracranial hypertension as its most prominent clinical features (Stam, 2005; Scheffer et al., 2017). The disease often affects young adults, women of childbearing age and children. Previously, the incidence was thought to be 0.2–0.5 per 100,000 person-years (Stam, 2005; Bousser and Ferro, 2007). However, more recent studies have shown that the incidence of CVT is higher than expected and may be as high as 1.32–1.57 per 100,000 person-years (Coutinho et al., 2012; Devasagayam et al., 2016). This difference may be the result of the use of more advanced diagnostic techniques. More importantly, CVT has received an increased amount of attention by clinicians. Patients with CVT often have headaches, seizures, altered consciousness, and neurological focal signs, all of which are non-specific manifestations, making it difficult to diagnose this disease. One study reported that 50% of CVT patients had a poor prognosis (Khealani et al., 2008). However, if an early correct identification of CVT is achieved, the patient should receive appropriate treatment, such as anticoagulation therapy, the reduction of intracranial pressure or neurological surgery. The majority of patients who are diagnosed and treated early have a generally good prognosis (Nasr et al., 2013; Sidhom et al., 2014; Terni et al., 2015; Kalita et al., 2016; Sassi et al., 2016; Lee et al., 2017).

## Search methods

**Figure 1:**
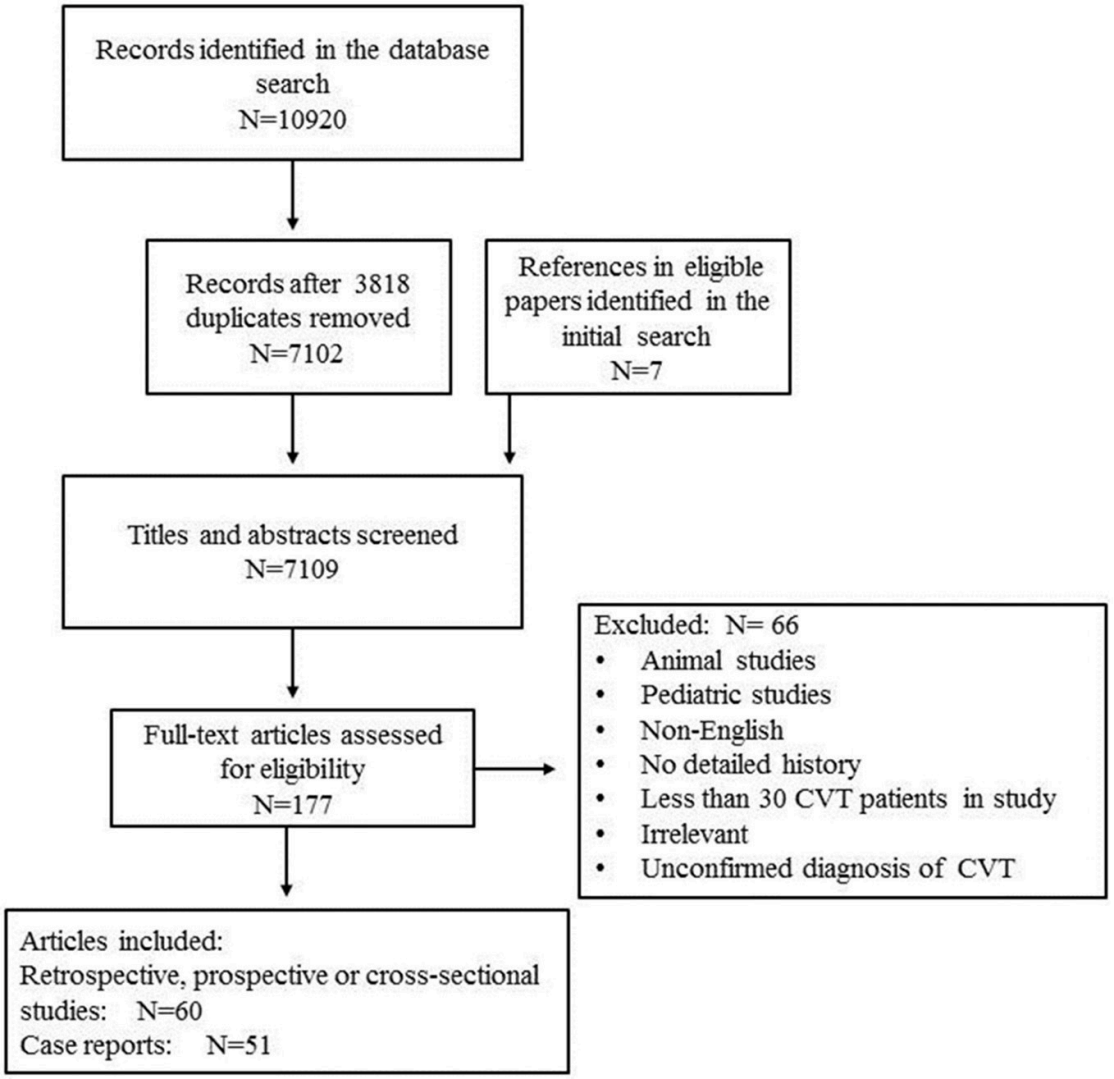
Flowchart of the article search. N, number of studies.

We performed a literature search in the PubMed, EMBASE, and Medline databases through to September 1, 2017. The following search query was used in PubMed: (“Sinus Thrombosis, Intracranial”[Mesh]) OR (sinus^*^[Title] AND thrombosis[Title]) OR (cerebral[Title] AND (venous[Title] OR vein^*^[Title]) AND thrombosis[Title]). An equipollent search query was used to search the EMBASE and Medline databases. The references in eligible papers identified in the initial search were also screened. To collect information related to older cases, the relevant books and monographs were also searched. The author reviewed the titles and abstracts of the papers identified in the search to make a preliminary evaluation of their eligibility. The full text of all potentially eligible papers was then accessed and read. Only when a paper met the eligibility criteria and was found to be relevant to this review was it included (see Figure 1). The following eligibility criteria were applied: (1) Only human studies were considered. (2) Because the clinical manifestations of childhood and neonatal patients are different, pediatric studies were excluded except for some relevant epidemiological studies. (3) Publications written in English or in other languages but with an English abstract that contained sufficient information were eligible. (4) All patients included in the study were diagnosed by surgery or an autopsy or based on an imaging examination, such as intra-arterial angiography (DSA), magnetic resonance imaging (MRI), MR angiography (MRA), MR venography (MRV), computed tomography venography (CTV), or CT angiography (CTA). All studies that contained patients with an unconfirmed diagnosis of CVT were excluded. (5) With regard for case reports, a detailed and reliable medical history, the results of a physical examination, or the results of laboratory and imaging examinations were required. (6) To minimize bias, retrospective and prospective studies were required to contain at least 30 patients diagnosed with CVT to be eligible, whereas case and case-series reports were not required to meet this limit. (7) Papers concerning the epidemiology, clinical manifestations, treatment, or prognosis of CVT were eligible.

## History

In 1825, Ribes (1825) described the first case of CVT, which was later confirmed by autopsy, in a 45-year-old male with severe headache, seizures, delirium, and thrombosis involving the superior sagittal and lateral sinuses. In 1828, Abercrombie (1828) reported the first postpartum CVT in a 24-year-old woman who developed headache and seizures 2 weeks after an unremarkable delivery. A subsequent autopsy revealed thrombosis of the superior sagittal sinus and cortical veins. Since these cases were published, many additional sporadic cases have been reported, and all of these were confirmed by autopsy. In rare cases, a diagnosis was achieved by angiography. Because it is difficult to diagnose, CVT is considered a serious and rare disease (Goldman et al., 1964; Rousseaux et al., 1977). In 1968, (Krayenbuhl, 1968) conducted a retrospective study of 92 patients with CVT diagnosed by angiography, surgery, or autopsy. Thirty-seven of these cases (40%) were associated with infection, and 17 of the remaining 55 non-infectious CVT cases were associated with pregnancy, abortion, or the perinatal period. In these patients, headache (60/92), varying degrees of paresis (56/92), and seizures (34/92) were the most common manifestations. Headache was often unilateral, and seizures were usually furious. Among these 92 patients, 35 (38%) died. In 1985, Bousser et al. (1985) found that the incidence of infectious CVT was significantly reduced by the use of antibiotics, whereas the incidence of CVT associated with other factors, including trauma, Behcet's disease, the perinatal period, the use of oral contraceptives, neoplasms, nephrotic syndrome, coagulation factor, and other abnormalities, was higher. With the advent of new diagnostic techniques, such as CTV and MRA, it has become easier to achieve an early diagnosis of CVT (Poon et al., 2007). Currently, the largest studies to explore CVT are an Italian multicenter study of 706 CVT patients and a multicenter, multi-country study of 624 patients (the International study on Cerebral Vein and Dural Sinus Thrombosis, ISCVT; Ferro et al., 2004; Dentali et al., 2012).

## Epidemiology

### Incidence

In 1995, studies estimated that the incidence of CVT was lower than 10 cases per million per year (Daif et al., 1995). In 2012, Coutinho et al. (2012) conducted a retrospective cross-sectional study and found that the incidence of CVT was higher than previously expected and perhaps as high as 13.2 cases per million per year. A large retrospective census conducted by Devasagayam et al. (2016) in 2016 also supported this conclusion. The incidence of CVT in neonates and children is ~0.67 cases per 100,000 children per year, and the incidence of perinatal CVT is ~11.6 cases per 100,000 deliveries in pregnant women (Lanska and Kryscio, 2000; deVeber et al., 2001).

### Gender

In 1985, it was found that the male to female ratio in CVT was 1:0.8 (Bousser et al., 1985). In two studies published in 2014 and 2016, there were 3.7–5.3 times more female than male patients (Karadas et al., 2014; Gunes et al., 2016). Zuurbier et al. (2016b) conducted a systematic analysis of the proportions of male and female patients in 23,638 patients across 112 studies published from 1966 to 2014. They found that the proportion of CVT patients who were female gradually increased over time and that the median proportion of female patients was 54.8% in studies published before 1981 and 69.8% in studies published after 2001. This difference was significant (*P* = 0.002), and the year of publication of the studies was associated with the proportion of patients who were female (*P* = 0.01). The proportion of female CVT cases that were pregnancy-related remained stable over time, whereas the proportion of affected patients using oral contraceptives increased over time (Pearson's correlation coefficient 0.29, *P* = 0.01). This may explain the observed increase in the proportion of female patients.

### Age

CVT often occurs in young people and women of childbearing age. Affected children are usually newborns. The age of CVT patients ranges from newborn to 82 years old, but this condition mainly occurs in individuals between 30 and 41 years old (Table 1; deVeber et al., 2001; Wasay et al., 2008; Sidhom et al., 2014; Kalita et al., 2016).

**Table 1:** The clinical features of CVT.

**General manifestation**	**References**	**Number of cases**	**Results**
Age	Wasay et al., 2008	182	13–82 years old (average, 38 years old)
	Sidhom et al., 2014	41	Average, 41.24 years old
	Kalita et al., 2016	86	Median age, 30 years old (range, 6–76 years old)
	deVeber et al., 2001	160 children with CVT	43% were newborns (<1 month old), and 54% were <1 year old
Gender	Bousser et al., 1985	38	17 females (44.7%), 21 males (55.3%)
	Daif et al., 1995	40	50% female, 50% male
	Hinnell et al., 2012	108	62% female, 38% male
	Karadas et al., 2014	51	43 (83.4%) females, 8 (15.7%) males
	Gunes et al., 2016	75	78.7% female
Incidence	Coutinho et al., 2012	Among all 19 hospitals located in 2 Dutch provinces serving 3.1 million people	1.32 per 100,000 person-years
	Lanska and Kryscio, 2000	170 cases of postpartum cerebral venous thrombosis in 1 408,015 extracted sampled deliveries	11.6 cases of peripartum intracranial venous thrombosis per 100,000 deliveries
	deVeber et al., 2001	160 children with CVT	0.67 case per 100,000 children per year
Mortality	Krayenbuhl, 1968	92 cases of CVT	38% of the patients died
	Wasay et al., 2008	182 cases of CVT	24 patients died, the total mortality rate was 13%
	Borhani Haghighi et al., 2012	3,488 cases of CVT	Overall mortality was 4.39%
	Nasr et al., 2013	11,400 inpatients diagnosed with CVT during 2001–2008	232 patients (2%) died. Mortality: 15–49 years old (1.5%), 50–64 years old (2.8%), 65 years old or older (6.1%).
Mode of onset	Terazzi et al., 2005	48 cases	Acute onset in 21 cases (44%), subacute onset in 17 cases (35%), chronic onset in 10 cases (21%)
	Sidhom et al., 2014	41 cases	Acute onset in 10 cases (24%), subacute onset in 26 cases (64%), chronic onset in 5 cases (12%).
Time from onset to diagnosis	Ferro et al., 2009	624 cases	The median time was 7 days and the interquartile range was 3 to 16 days.
	Mahale et al., 2016	100 cases	Average, 7.6 ± 11.1 days; range, 1–60 days
Location	Sidhom et al., 2014	41 cases	Lateral sinus (56%) and superior sagittal sinus (51%) were most frequently involved. Forty-six percent of patients exhibited multiple venous sinus involvement.
	Uzar et al., 2012	47 cases	The sigmoid sinus was involved in 35 patients (74.5%), the transverse sinus was involved in 29 (61.7%) patients, and the superior sagittal sinus was involved in 21 cases (44.7%).
	Sassi et al., 2016	160 cases	The most common locations of thrombosis were the superior sagittal sinus (65%) and transverse sinus (60.5%). One hundred and fourteen (71.2%) patients exhibited multiple venous sinus involvement.
Prognosis	Ferro et al., 2004	624 cases	At the end of the follow-up (median, 16 months), 57.1% had modified Rankin Scale (mRS) = 0, 22% had mRS = 1, 7.5% had mRS = 2, 2.9% had mRS = 3, 2.2% had mRS = 4 or 5, and 8.3% had died.
	Hiltunen et al., 2016	161 cases	84% of the patients had a mRS of 0-1, and 42% had residual symptoms.
Cause of death	Canhão et al., 2005	624 cases	Transtentorial hernia (20/27) was the leading cause of death, and other causes of death included cardiopulmonary arrest, sudden death, pulmonary embolism, and sepsis.
	Ferro et al., 2001	142 cases	Cerebral edema with or without seizures (7/9) was the main cause of death. Others include anoxia secondary to a seizure and sudden cardiopulmonary arrest.

### Location

In the majority of patients, multiple venous sinuses are involved. The superior sagittal sinus, lateral sinus, and sigmoid sinus are most frequently involved. Sassi et al. (2016) conducted a retrospective study of 160 patients diagnosed with CVT. The most common thrombosis sites were the superior sagittal sinus (65%) and the transverse sinus (60.5%). In most patients, multiple venous sinuses (114 cases, 71.2%) were involved. In a study conducted by Sidhom et al. (2014), the most frequently involved sinuses were the lateral sinus (56%) and the superior sagittal sinus (51%), and in nearly half of all affected patients, multiple venous sinuses were involved. Uzar et al. (2012) conducted a retrospective study of 47 patients. The sigmoid sinus was involved in 35 (74.5%), the transverse sinus was involved in 29 (61.7%), and the superior sagittal sinus was involved in 21 (44.7%) of these patients. A multicenter, multi-country study reported that ~10.9% of patients with CVT exhibit deep venous system involvement, 17.1% exhibit cortical vein involvement, and 11.9% exhibit jugular vein involvement. The cerebellum and cavernous sinus were involved in only 0.3 and 1.3% of the patients, respectively (Ferro et al., 2004).

## Clinical features

### Disease onset

Approximately 80% of CVT cases have an acute to subacute onset. The median time from onset to diagnosis is ~7 days. When a patient is admitted to the hospital with altered consciousness, mental disorders and seizures, a diagnosis is obtained earlier (Ferro et al., 2009). Approximately 20% of patients had a chronic onset. Chronic CVT patients require extra attention to avoid a misdiagnosis because of the presentation associated with its untraditional onset.

### Headache

Headache is the most common manifestation of CVT and is observed, to varying degrees, in 80–90% of patients (Agostoni, 2004; Gunes et al., 2016). In CVT, 80.4–84% of headaches are acute to subacute, and a few are chronic headaches (Wasay et al., 2010; Uzar et al., 2012). A CVT-associated headache is generally persistent (77.8–86.9%; Iurlaro et al., 2004; Sparaco et al., 2015) and is positively related to the severity of the disease. If a chronic intermittent headache becomes aggravated or a new chronic headache appears, the possibility of CVT should be taken into consideration (Iurlaro et al., 2004; Uzar et al., 2012). Zhang et al. (2011) reported 2 CVT patients with chronic headache. In both patients, the headache was at first paroxysmal, light and self-limited but then gradually developed into a persistent severe headache. In CVT, a unilateral or localized headache is observed in ~65.2–66.7% of cases (Sparaco et al., 2015). In most cases, the headache is not related to the site of venous sinus thrombosis, but some studies have suggested that one exception is the sigmoid sinus thrombosis (Wasay et al., 2010; Timóteo et al., 2012; Sparaco et al., 2015). A retrospective case analysis published by Wasay et al. (2010) found that 17/28 (61%) patients with thrombosis involving only the sigmoid sinus or both the sigmoid and transverse sinus manifested occipital or neck pain. The properties of CVT headaches are diverse. They can manifest as throbbing, band-like, or burning or as thunderclap and other types of headache. Wasay et al. (2010) analyzed 200 patients with CVT and found that of the 72 who reported a headache, it was throbbing in 12 (9%), band-like in 27 (20%), thunderclap in 7 (5%), and had other properties (e.g., pounding, exploding, or stabbing) in 26 cases (20%). Sparaco et al. (2015) also reported the following properties of CVT headaches: 69.5% of the patients reported a throbbing headache, 8.7% a burning headache and 21.7% a tightening headache.

#### Thunderclap headache

A thunderclap headache is noteworthy. These headaches are a serious headache that occurs suddenly and reaches a peak within 1 min. It can be seen in various diseases, the most well-known of which is subarachnoid hemorrhage. In CVT, a thunderclap headache can be combined with or without subarachnoid hemorrhage. Studies have shown that 5–13% of CVT patients may have a thunderclap headache (Wasay et al., 2010; Sparaco et al., 2015). de Bruijn et al. (1996) reported that 10 out of 71 patients with CVT had a thunderclap headache as a prominent manifestation. These included five cases who had a normal CT scan at admission, three cases with subarachnoid hemorrhage, and two with multiple cerebral hemorrhage. The authors suggest that it is difficult to distinguish a thunderclap headache in CVT from a headache caused by subarachnoid hemorrhage because the properties, clinical symptoms, and signs of the headaches are similar. Coutinho et al. (Coutinho and Stam, 2011) reported a 44-year-old female who suddenly had 2 days prior had a serious headache that lasted for a few seconds while she was wake. The headache was diffuse, radiated to the neck, and was accompanied by nausea, vomiting, and photophobia. A physical examination was unremarkable except that she exhibited mild neck resistance. A CT scan showed a hyperdense aspect of the straight sinus and no signs of subarachnoid hemorrhage. Further examination revealed complete thrombosis of the straight sinus and partial thrombosis of the bilateral transverse sinus. After the patient underwent 1 month of anticoagulation therapy, she fully recovered. Hassan et al. (2015) reported a 35-year-old male in whom the condition manifested mainly as a severe headache and partial seizure. A physical examination showed mild bilateral papilledema and neck resistance. A cerebral CT showed bilateral subarachnoid hemorrhage and complete thrombosis of the superior sagittal sinus. An MRV also indicated that the superior sagittal sinus was completely occluded. The patient's symptoms were relieved after he underwent anticoagulation therapy for 1 week.

#### Migraine-like headache

A CVT-related headache can manifest as unilateral and pulsatile, similar to a migraine (with or without aura). Sparaco et al. (2015) conducted a case series study that included 25 patients who were diagnosed with CVT. Of these patients, 23 had headaches and 16/23 (69.5%) patients with headache met the diagnostic criteria for migraine headaches. Tan et al. (2015) reported a case involving a 20-year-old female who started taking contraceptives for polycystic ovary syndrome 3 months before the onset of CVT. She had a severe unilateral throbbing headache that was aggravated by physical activity and accompanied by photophobia. A CT was normal, and she was diagnosed with a migraine for the first time. However, triptan treatment was ineffective, and the headaches persisted for more than 72 h. Further MRV indicated a thrombosis in the left sigmoid sinus. Slooter et al. (2002) reported a CVT patient who was first misdiagnosed with migraine with aura. This 24-year-old female patient initially experienced whole body discomfort, such as palpitations, fatigue, and nausea, for ~1 h. Then, she gradually developed tingling and numbness of the left side of her body that lasted for half an hour, and she later felt weakness in her left limbs for half an hour. She then immediately had a serious pulsatile headache on the right side, and during this time she was less alert but could still answer questions. Approximately 4 h after the headache disappeared, she felt tired and had dry mouth and frequent micturition. Her physical examination and previous medical history were unremarkable except for tension-type headaches. Therefore, this headache was considered to be the first attack of a migraine with aura. Similar episodes occurred 3 times over 5 days, and a further MRV revealed a thrombosis of the right lateral sinus.

#### Cluster headache

A CVT-related headache can also mimic a cluster headache. Cluster headache refers to sudden, serious, and unilateral periocular pain that is often recurrent and lasts for 15–180 min [Headache Classification Committee of the International Headache Society (IHS), 2013]. Cluster headaches are usually associated with brain autonomic symptoms and anxiety. Georgiadis et al. (2007) reported on a 46-year-old male smoker who had no history of headache or other neurological disease before the onset of a severe paroxysmal headache 3 weeks before the visit. The most painful part of the headache was in the left temporal region. The headache was accompanied by left postocular tingling and left-side lacrimation and rhinorrhea. At first, the headache lasted for an hour, during which patient was agitated and only calmed down when the headache was relieved. The headache initially occurred at 23:00 every night and then became more frequent. Orally administering acetyl salicylic acid (1,000 mg) 15 min before the onset of the headache shortened the attack to 15 min. Inhaling pure oxygen was also effective. MRI and MRV examinations suggested a venous sinus thrombosis in the superior sagittal and bilateral transverse sinuses. Rodríguez et al. (2008) reported a 51-year-old male who developed severe paroxysmal unilateral headaches that lasted for up to 90 min. The headaches were located around the right eye, which exhibited conjunctival congestion, lacrimation, ptosis and miosis. The frequency of the headaches was ~10 times per day. The patient was given ergotamine (2 mg) or zolmitriptan (5 mg), which alleviated the headaches. A neurological examination and cerebral CT were normal, and there was no bilateral papilledema. The headache disappeared 4 weeks later. Three months later, the patient was re-admitted as a result of bilateral partial motor seizures and headache lasting 24 h. A physical examination revealed mild left-side hemiparalysis and a bilateral plantar extensor response. Cerebral MRI and MRV revealed a venous thrombosis of the superior sagittal and bilateral transverse sinuses. The patient was discharged after undergoing anticoagulation treatment and was followed up for 18 months without headache. Other authors have reported similar findings (Peterlin et al., 2006; Bellamio et al., 2017).

#### Post-dural puncture headache

Similarly, post-dural puncture headache (PDPH) has also been reported in CVT. After lumbar puncture, if the headache is sustained or progressively aggravated or new symptoms appear, especially in patients with CVT susceptibility factors, the possibility of CVT should be considered. Guner et al. (2015) conducted a retrospective study of 46 patients diagnosed with CVT. Of these, 19.6% (9 cases) had a lumbar history before onset. The authors suggested that patients with persistent or progressive headache after lumbar puncture should be reassessed. Sherfudeen et al. (2016) described a 33-year-old male who underwent fissurectomy under spinal anesthesia and had no remarkable discomfort after surgery. The patient was discharged the next day. On the second day after discharge, the patient developed a headache that was diffuse, aggravated in an upright position and relieved in a supine position. The headache was not associated with nausea, vomiting, or other signs of nervous system impairment. He was diagnosed with PDPH and prepared for an autologous blood patch (AEBP). During the AEBP, the patient went into respiratory arrest and lost consciousness. After a few minutes of mask ventilation, the patient regained consciousness without neurological deficits. During the next 24 h, the headache was only slightly alleviated, and thereafter, the headache became further aggravated and was worse in an upright position. Further MRI identified thrombosis of the superior sagittal sinus and bilateral large cortical vein. Ghatge et al. (2008) described a 22-year-old pregnant woman who had an unsuccessful epidural anesthesia during delivery so the intravenous anesthesia was chosen instead. This patient developed a frontal and occipital headache 24 h after delivery that was relieved by lying down. No remarkable neurological signs were found. She was diagnosed with PDPH and underwent AEBP the next day. Forty-eight hours later, the headache had eased, but the site of the headache had moved to the forehead and neck. She was readmitted as a result of a headache 3 days after discharge. The headache was located in the middle of the forehead and was not related to position, and the patient complained that she could not feel her legs and had transient difficulty maintaining balance. A neurological examination was normal, but 12 h later, the patient developed confusion, dysphasia, right limb weakness, lethargy and vomiting. CVT suggested thrombosis of the vein of Galen, the straight sinus and the posterior third of the superior sagittal sinus.

CVT headaches are usually accompanied by other manifestations, such as seizures, altered consciousness and focal neurological deficits. However, in 14–40% of patients, headache is the only symptom (Cumurciuc et al., 2005; Timóteo et al., 2012). Cumurciuc et al. (2005) conducted a study in which they selected 17 patients from 123 CVT patients who presented with isolated headaches but had normal CT and cerebrospinal fluid (CSF) examinations. Sixty-five percent (11 cases) of the patients had progressive exacerbated headaches, 17.5% (3 cases) had acute headaches, and 17.5% (3 cases) had thunderclap headaches. Four of the patients had a history of migraine without aura before the onset of CVT. Among these four patients, three had headaches that were different from their previous headaches, and one patient had headaches that shared the same properties as but were more persistent than previous headaches. Forty percent of the patients had a normal CT. The authors concluded that all patients who develop new headaches (progressive or thunderclap) but had a normal CT and CSF, MRI/MRV examinations should be performed. Timoteo (Timóteo et al., 2012) conducted a study that included 30 patients who were diagnosed with CVT, and they selected 12 patients with isolated headaches who also had a normal neurological examination, a normal optic disc, and no hemorrhage or parenchyma injury on CT. No uniform type of headache was experienced by these patients except that their headaches were bilateral, and 7 patients complained that sleeping, lying down, performing a Valsalva action or exerting themselves increased the severity of the headache. The diagnostic time was substantially delayed in patients who had only headaches. The authors suggested that attention should be paid to patients with the following types of headaches: recently persistent headache, thunderclap headache or headaches aggravated by sleeping, lying down, a Valsalva action or exertion, even if there is neither papilledema nor focal signs. In these patients, CVT should be considered. Unal et al. (2016) described a patient with CVT who was misdiagnosed with subarachnoid hemorrhage. This 78-year-old male had intermittent headache 1 year before the onset of CVT and was admitted as a result of the sudden onset of a severe headache. The properties of the headache were different that those of previous headaches. Headache was the only manifestation. He had a history of stroke 1 year prior and was taking the long-term medication clopidogrel. A neurological examination was normal. MRV suggested venous sinus thrombosis involving the straight sinus, superior sagittal sinus, bilateral transverse sinus and sigmoid sinus. He was discharged after anticoagulation therapy, and the headache had completely disappeared at a follow-up 2 weeks later.

Some patients with CVT have no headache, and these patients are usually older and are less likely to be female. In patients without headache, paralysis, and seizures are more common, while papilledema is rare. Isolated cortical venous thrombosis, parenchymal lesions, and malignant diseases are also more common in patients without headache (Coutinho et al., 2015).

### Seizures

Seizures are one of the common symptoms of CVT and are found in ~40% of CVT patients (Masuhr et al., 2006; Ferro et al., 2008; Mahale et al., 2016). According to the type of seizure, generalized seizures are the most common, followed by focal seizures, and some patients have both types of seizures. Mahale et al. (2016) studied seizures in CVT and found that 46% (46/100) of patients with CVT had acute seizures. The patients with seizures were could be separated into focal (*n* = 4), focal with secondary generalized (*n* = 9), and generalized tonic clonic (*n* = 33) seizures. The focal seizures were simple partial seizures and no complex partial seizures with or without generalization were observed. With regard for frequency, 7 patients (15.2%) exhibited generalized convulsive status epilepticus, 22 (47.8%) had clustered seizures, and 17 (37%) had a single seizure episode. All patients with seizures (46 patients) were administered antiepileptic drugs (AEDs), and 10 patients without seizures also took AEDs. The remaining 44 patients without seizures did not take AEDs. Forty-four patients with seizures were treated with monotherapy, and 2 patients required two AEDs to control their seizures. The authors found that the predictors of acute seizures included altered consciousness (Glasgow Coma Score, GCS < 8), focal injury, hemorrhagic infarction, frontal lobe involvement, superior sagittal sinus thrombosis, and high levels of D-dimer.

Masuhr et al. (2006) defined early symptomatic seizures in CVT as seizures that occurred from the onset of clinical symptoms to 14 days after a diagnosis of CVT and before any AEDs were used. They conducted a retrospective case study to determine the risk factors and predictors of early epileptic seizures in CVT. During the acute phase, 86 (44.3%) out of 194 patients with acute CST developed early seizures. Among the patients with seizures, 65 (75.6%) had secondary generalized seizures, 21 (24.4%) had focal seizures, and 47 (54.7%) had Todd's paralysis. Continuous secondary generalized seizures or focal motor seizures (defined as more than or equal to 3 in 24 h) and status epilepticus were observed in 19 patients (22.1%) and 11 patients (12.8%), respectively. The authors found that movement disorders, intracranial hemorrhage, and cortical vein thrombosis were the most important risk factors for early seizures. In these patients, prophylactic medication with AEDs may be an option. Ferro et al. (2008) conducted a multicenter prospective observational study in which they analyzed early symptomatic seizures in CVT. They divided early symptomatic seizures into early seizures and presenting seizures. Presenting seizures were defined as cases in which the onset of seizures occurred at or before a diagnosis of CVT, while early seizures were defined as seizures that were experienced after a diagnosis of CVT. Their study included 624 patients, 245 (39.3%) of whom had presenting seizures. Among patients with presenting seizures, 58 (23.7%) had focal seizures, 123 (50.2%) had generalized (primary or secondary) seizures, and 64 had two type of seizures (26.1%). Three patients (0.5%) had status epilepticus. Two patients had a history of complicated partial epilepsy in which the seizures were a different type. Forty-three patients (6.9%) had early seizures, and 60% of these (26/43) had recurrent episodes of seizures. In other words, these patients had both early and presenting seizures. Two patients with previous epilepsy had no early seizures. The authors found that CVT patients with supratentorial lesion involvement had a higher risk of both early and presenting seizures. There was a higher risk of early seizures occurring within 2 weeks in patients with presenting seizures. The incidence of early seizures was lower when AED prophylaxis was used in patients with supratentorial lesions and presenting seizures. The authors suggested that it may therefore be reasonable to use prophylactic AEDs in acute CVT patients with seizures and supratentorial involvement. Kalita et al. (2012) also found that supratentorial lesions were associated with a higher risk of seizures.

Seizures in CVT can manifest as single or cluster episodes. Status epilepticus is observed in 5.6–7% of CVT patients (Masuhr et al., 2006; Mahale et al., 2016). Although status epilepticus is a vital neurological emergency, its impact on mortality is unclear. Studies have shown that among CVT patients with seizures, mortality was 3 times higher in patients with status epilepticus (36.4%) than in those without status epilepticus (12%; Masuhr et al., 2006). However, different results have also been published. Mahale et al. (2016) conducted a retrospective study of 46 patients with CVT with seizures, including 7 patients with status epilepticus, and none of the patients died, whereas among the 39 patients without status epilepticus, 4 (10.3%) died. Kalita et al. (2012) conducted a retrospective case study of 42 CVT patients with seizures. Al1 10 patients with status epilepticus survived, whereas of the 32 patients without status epilepticus, 5 (15.6%) died. The authors suggested that seizures are not associated with death and 6-month outcome.

### Focal neurological deficits

Some CVT patients present with neurological deficits, including motor/sensory impairment, aphasia, cranial nerve palsy, and cortical blindness, as the main manifestation. Neurological focal lesions are associated with larger cerebral infarctions involving the proximal midline of the Rolandic region, the posterior temporal region, the frontal-parietal and the parietal-temporal region. Focal neurological deficits are more common in non-inflammatory CVT, and cavernous sinus syndrome is more common in infection-related CVT (Paciaroni et al., 2008; Korathanakhun et al., 2015).

Motor deficits were the most common neurological deficits and were observed in 19.1–39% of CVT patients. Motor deficits were more common in CVT patients with superior sagittal sinus, cortical veins, and cerebral deep venous system involvement. Studies have shown that almost the same proportion of left and right limb involvement is observed. Motor deficits in CVT can be similar to those in arterial stroke, including a sudden onset, and they can also be similar to those in an intracranial space-occupying lesion with subacute onset. A very small number of patients can manifest symptoms like those observed in a transient ischemic attack (TIA; Ferro et al., 2004; Bousser and Ferro, 2007; Paciaroni et al., 2008; Wasay et al., 2008; Uzar et al., 2012). Manzano Palomo et al. (2006) reported a 40-year-old male who showed left limb paresis and recovered completely within the several minutes. MRA suggested thrombosis of the superior sagittal sinus, the bilateral transverse sinus and the right side of the sigmoid sinus in addition to thickening of the veins on the brain surface that reflux to the left sigmoid sinus. The patient was diagnosed with chronic CVT. Many studies have found that hemiparesis is associated with a poor prognosis (Uzar et al., 2012; Nasr et al., 2013).

Aphasia is also a common neurological defect that is observed in 19–24% of CVT patients (Ferro et al., 2004; Sparaco et al., 2015). Aphasia is commonly observed in left lateral sinus thrombosis or deep vein thrombosis (Bousser and Ferro, 2007). Damak et al. (2009) conducted a study of 157 patients with lateral sinus involvement out of 195 CVT patients. Among these, 62/195 patients (32%) who had isolated lateral sinus involvement were included and compared with the other 133 patients. In all, 19 patients (31%) had at least one focal sign (focal deficits or partial seizure), and dysphasia was the most common focal sign (8 cases). Tuncel et al. (2015) reported a 27-year-old male who developed CVT with motor aphasia due to thrombosis of the left lateral sinus, and he fully recovered within 1 month of anticoagulation therapy. Ferro et al. (2002) found that no aphasia was one of the predictors of a complete recovery.

Ameri (Ameri and Bousser, 1992) reported that 12% of CVT patients had cranial nerve involvement, and III, IV, V, VI, VII, VIII, IX, X, and XII cranial nerve palsy has been reported. Cranial nerve involvement can be multiple or single, and in some CVT cases, cranial nerve involvement is the only manifestation (Paciaroni et al., 2008). Patients with CVT may have isolated diplopia. Panos et al. (2014) reported a case of a 23-year-old female who was admitted to the hospital because of intermittent diplopia that had lasted 3 weeks. A physical examination showed oculomotor paralysis and no headache. She was first misdiagnosed as optic neuritis, and further MRI and MRV resulted in a diagnosis of CVT. Patients with CVT may also have isolated VI nerve palsy. Mittal et al. (2017) reported a case of isolated ipsilateral VI nerve palsy that was caused by unilateral inferior petrosal sinus thrombosis. The patient did not have cerebrovascular disease risk factors and manifested as binocular horizontal diplopia without exhibiting increased intracranial pressure. A physical examination found that the left abducens nerve was completely paralyzed, and CSF tests and MRI were normal. MRV revealed thrombosis of the left internal jugular vein, left sigmoid sinus and left lateral sinus. The cavernous sinus was normal, and no mastoiditis was found. Similarly, Sotoodehnia et al. (2016) reported a case of isolated abducens nerve palsy that was caused by left transverse sinus thrombosis. In CVT, when the V nerve is involved, symptoms can manifest as trigeminal neuralgia. Tsimiklis et al. (2014) reported a 33-year-old female with a history of superior sagittal sinus thrombosis characterized by severe recurrent trigeminal neuralgia. An examination revealed that this neuralgia was caused by varicose veins that had compressed the trigeminal nerve. When the VII nerve is involved, patients may exhibit peripheral facial paralysis. Straub et al. (2000) reported a 17-year-old female who developed isolated right facial paralysis 2 weeks after the onset of symptoms. MRV suggested that the ipsilateral transverse sinus vein was partially occluded. After recanalization of the transverse sinus was performed, the facial nerve paralysis was completely recovered. A facial neurography had excluded idiopathic palsy and suggested a proximal conduction block to the facial canal. Other authors have reported similar cases of facial nerve paralysis in CVT (O'Connor et al., 2015).

CVT can also cause multiple cranial nerve paralysis. Topsakal et al. (2002) reported a case of 27-year-old female CVT patient that mainly manifested as multiple cranial nerve paralysis with TIA. She had headaches and right peripheral facial paralysis 25 days before the visit and left peripheral facial paralysis from 2 days before the visit. During the course of her recovery, she was admitted because of sudden horizontal and vertical diplopia. A physical examination revealed right side IV and VI cranial nerve paralysis and papilledema and right orbital venous congestion. On the first day of admission, seizures appeared. MRI and MRV suggested complete thrombosis of the superior and inferior sagittal sinus and significant collateral venous channels, but no parenchymal lesion was observed. The IV and VII cranial nerve paralysis and left hemiplegia completely recovered within 2 days, but a 30-min episode of right hemiplegia occurred. After treatment, two venous sinuses were recanalized. She was discharged after a 40-day hospitalization with only slight right abducens nerve paralysis. Mubbashir Shariff et al. (Mubbashir Shariff and Alhameed, 2014) reported a 19-year-old boy with extensive CVT after whiplash injuries. The CVT manifested as right facial nerves paralysis and ipsilateral venous sinus thrombosis. Left facial nerve paralysis and partial left occulomotor weakness followed. Posterior group cranial nerves can also be involved in CVT. Byju et al. (2012) reported a case of a pregnant woman who showed right sensory nerve deafness and IX, X, and XII cranial nerve paralysis on the right side. She was diagnosed with thrombosis of the right transverse sinus and sigmoid sinus stretching into the deep jugular vein and torcular herophili, and her symptoms improved after anticoagulation treatment.

### Neuro-ophthalmological symptoms

CVT can also cause neuro-ophthalmological symptoms, such as papilledema, loss of vision, and constriction of the visual field. Papilledema is a common manifestation of CVT that is observed in 28–67.5% of CVT patients. Papilledema is rare in CVT patients without headache (Ferro et al., 2004; Wasay et al., 2008; O'Rourke et al., 2014; Coutinho et al., 2015; Thammishetti et al., 2016). In patients with chronic onset or a late visit, papilledema is more frequent than in patients with acute onset (Ferro et al., 2005). Coutinho et al. (2014) found that papilledema was more common in patients with cortical hemorrhage (44 vs. 9%) than in those without. Eliseeva et al. (2015) conducted a retrospective case study of 49 patients diagnosed with CVT, including 15 who were acute (30.6%), 11 who were subacute (22.4%) and 23 who were chronic (47.0%). Papilledema was observed in 84.6% of the CVT patients with acute and subacute onset, and all patients with chronic onset had papilledema. Of the patients with acute and subacute onset, only 4% had visual impairment, and 65.2% of patients with chronic onset had visual impairment caused by papilledema or optic atrophy after papilledema.

In CVT patients, visual impairment is observed in 13.2% of patients (Ferro et al., 2004). Visual impairment can be caused by papilledema, which is associated with intracranial hypertension, a condition in which vision impairment occurs relatively slowly. Visual impairment can also be caused by focal lesions, including cerebral infarction and cerebral hemorrhage, and the onset of visual damage is usually obvious and rapid. Acute visual impairment often leads to cortical blindness, which can be caused by CVT involving the geniculocalcarine tract, especially the primary visual cortex (Das and Huxlin, 2010). In CVT, acute visual loss usually manifests as bilateral homonymous hemianopsia and sometimes as total blindness. This loss of vision is often reversible, to some extent, and while patients may have residual visual field defects, sometimes vision is fully restored. Wang et al. (2013) reported a case of an 18-year-old female who had a history of headache for 3 weeks and developed acute bilateral blindness after a nap. Both pupils and an ophthalmoscopy were normal, and both eyes showed no light perception. MRV suggested that the left transverse sinus and sigmoid sinus were completely occluded, and the posterior superior sagittal sinus was also nearly occluded. She was initially treated with intravascular mechanical thrombolysis, and systemic anticoagulation and vascular recanalization were later achieved. Her left visual field was recovered, but exhibited right homonymous hemianopsia when she was discharged. Ko et al. (2001) reported a case of a 54-year-old female whose main manifestations were sudden severe headaches and progressive bilateral loss of vision within 1 day. She was conscious when admitted, and there was no abnormality on a neurological examination. An ophthalmic examination found that the corrected visual acuity of the right eye was 1/60 and that the left eye could only manage counting fingers. Binocular movement was normal. She had an isocoria of 4 mm, and her light reflexes were slow. A visual field examination suggested left homonymous hemianopsia. A cerebral CT showed a hematoma on the right side of the occipital temporal area and significant brain edema with a left-shifted midline. MRV suggested thromboses of the rear half of the superior sagittal sinus and adjacent cortical veins. At 3 weeks after anticoagulation therapy, the visual acuity of this patient was recovered to 6/6 in both eyes, but visual field defects persisted. Ray et al. (2013) reported a 47-year-old female who manifested with recurrent vomiting after a surgery and a progressively increasing headache in the occipital area. She developed acute bilateral painless loss of vision and asymmetric limb weakness 2 days later. A visual examination visual acuity in both eyes decreased to perception of light. The light reflexes of the bilateral pupils and bilateral eye movements were normal. Indirect ophthalmoscopy revealed mild to moderate edema of the right optic disc and mild edema of the left optic disc. MRI revealed a hyperintensity involving the bilateral parieto-occipital corticomedullary junction. MRV suggested superior sagittal sinus thrombosis. The patient's symptoms were completely relieved at 6 months after anticoagulation therapy. Bilateral occipital infarction induced by CVT can rarely cause a loss of vision and this manifestation suggests that CVT should be taken into account when acute painless visual loss is caused by occipital vascular lesions. Anticoagulant therapy can significantly improve the prognosis in these patients. Mitaki (Mitaki et al., 2008) also reported a similar patient with CVT with bilateral occipital lesions and cortical blindness.

Chronic visual loss is usually caused by secondary optic atrophy resulting from long-term papilledema. This kind of visual impairment is usually irreversible. Messouak et al. (2007) reported a case of a 20-year-old female who was admitted for intracranial hypertension. She had experienced a decrease in bilateral visual acuity over the past 2 months. A physical examination showed papilledema that was not accompanied by focal neurological signs. An imaging examination suggested superior sagittal sinus and lateral sinus thrombosis. After anticoagulation therapy and anti-edema treatment, the intracranial hypertension gradually recovered. However, because there was optic nerve atrophy, visual impairment persisted. O'Connor et al. (2015) reported a CVT patient with facial nerve paralysis who developed severe bilateral papilledema after 3 months because the patient was misdiagnosed. The facial nerve paralysis was substantially recovered after treatment, while severe visual impairment remained, perhaps because of optic atrophy-induced papilledema.

Visual symptoms related to CVT include vision loss, visual field defects and other negative symptoms, and CVT has also been reported to manifest as migraine-like visual phenomenon, such as colored photopsias, dark spots, and visual blurring associated with vertical wavy lines. Additionally, CVT patients have been reported to manifest with painful ophthalmoplegia and eye swelling (Newman et al., 1989; Napon and Kabore, 2010; Sakaida et al., 2014).

### Altered consciousness

Altered consciousness is observed in 20–30.6% of patients with CVT (Ferro et al., 2004; Wasay et al., 2008; Uzar et al., 2012; Sassi et al., 2016). Sassi et al. (2016) conducted a retrospective study in which 49 of 190 CVT patients had altered consciousness. Of the patients with altered consciousness, 28.6% (14/49) had mild altered consciousness (GCS10-15), 63.3% (31/49) had moderate altered consciousness (GCS8-10 points), and only a very small number of patients (4/49) had severely altered consciousness (GCS 3-7).

Patients with deep venous system thrombosis often experience altered consciousness (Bousser and Ferro, 2007). Terazzi et al. (2005) found that 61.5% of patients with deep vein CVT but only 17.1% of patients with cortical CVT had altered consciousness or confusion. Pfefferkorn et al. (2009) conducted a retrospective case study of 32 patients with deep venous thrombosis. In these patients, headache (81%) and a decreased level of consciousness (72%) were the most common symptoms, and they were usually accompanied by focal neurological deficits and neuropsychiatric manifestations, such as confusion and amnesia. Of the 32 included patients, 72% (23 cases) had a GCS ≤ 14, whereas 38% (12 cases) had a GCS ≤ 8. Four patients had a “hyperacute” course, meaning they had a GCS score ≤ 8 within 24 h of onset. In the deep venous system, all patients exhibited internal jugular vein involvement, and it was bilateral in 91% of these patients. Patients with straight sinus and vein of Galen involvement also accounted for 97 and 88% of the patients, respectively. The most common parenchymal lesion observed on CT or MRI was thalamic edema (69%), which was bilateral in 47% of patients. In all, 75% of the patients were stabilized and showed improvement after anticoagulation, while 25% of the patients worsened into a progressive coma within 6–48 h, and 6% of the patients died.

When a patient exhibits altered consciousness, a diagnosis may be made earlier, but altered consciousness is also closely related to a poor prognosis (Ferro et al., 2004; Pfefferkorn et al., 2009; Sassi et al., 2016). Kowoll et al. (2016) conducted a multicenter retrospective study of 114 CVT patients treated in an intensive care unit who had GCS ≤ 9. Among these patients, up to 44.7% were severely disabled (defined as mRS ≥ 4) and/or died. Uzar et al. (2012) conducted a retrospective study in Italy that included 47 patients. Twenty-five percent of the patients had altered consciousness, and acute onset, altered consciousness, hemorrhagic infarction, and hemiplegia were associated with increased mortality. Wasay et al. (2008) conducted a multicenter study in the United States that found that in CVT, coma and intracranial hemorrhage at admission were poor prognostic indicators. Some scholars have suggested that the 3-month prognosis in CVT patients is associated with GCS (Kalita et al., 2016).

### Other manifestations

CVT can also manifest as psychological symptoms. Hassan et al. (Hassan and Kumar, 2013) reported a 45-year-old male with headache, vomiting, and psychological symptoms at high altitudes who was diagnosed with deep venous thrombosis. He was abulic and uncooperative. MRI revealed venous cerebral infarctions in the bilateral thalamus, left basal ganglia and periventricular white matter. MRV suggested thromboses of the internal cerebral vein, septal veins, thalamostriate veins, vein of Galen and proximal portion of straight sinus. Xue et al. (2013) and Kaaniche et al. (2015) also reported a series of CVT patients who mainly manifested with psychiatric symptoms. A small number of CVT patients have subarachnoid hemorrhage. Sahin et al. (2014) reported a 48-year-old female who first manifested with subarachnoid hemorrhage and a thrombosis of the superior sagittal sinus. The authors performed a literature review of 73 cases of CVT who manifested as subarachnoid hemorrhage, and they found that CVT-related subarachnoid hemorrhage is usually located on the convex surface of the brain. Hassan et al. (2015) reported two CVT patients who had subarachnoid hemorrhage involving the sulci of the convexity of the hemisphere. Other scholars have published similar reports (Jaiser et al., 2008; Arévalo-Lorido and Carretero-Gómez, 2015). Most patients have a good prognosis after anticoagulation treatment. Therefore, in patients with subarachnoid hemorrhage, if the patient has susceptibility factors for CVT, CVT should be considered. CVT involving the cerebellum is very rare. Only 0.3–1.8% of CVT patients have cerebellum involvement, and the most common manifestations in these patients are headache, nausea, encephalopathy, ataxia and papilledema. The mortality rate in this group could be as high as 33% (Ferro et al., 2004; Kulkarni et al., 2014). CVT can also be expressed as transient global amnesia (TGA). Sharma et al. (2015) reported a 58-year-old male characterized by TGA. The patient suddenly exhibited anterograde amnesia accompanied by repeated questioning. He had impaired immediate and recent memory, and orientation to time was not intact. MRV suggested thrombosis involving the superior sagittal sinus and the torcular herophili. Patients with CVT can have a paroxysmal loss of consciousness, which can manifest as not only seizures but also syncope. García et al. (2013) reported a case of chronic CVT that showed recurrent syncope. The case was a young man who had two previous histories of venous thrombosis, superior vena cava thrombosis, and cerebral venous thrombosis (CVT). Several years after a lumboperitoneal shunt was performed for increased intracranial pressure, the patient had recurrent, frequent, transient dizziness followed by loss of consciousness. Simultaneous video electroencephalogram and electrocardiogram were normal. Cerebral angiography suggested chronic CVT, and lumbar puncture revealed that intracranial pressure was only 47 mmHg. The patient did not exhibit syncope after the lumboperitoneal shunt was replaced. A small number of CVT patients with lateral sinus involvement exhibit acute ipsilateral hearing loss as the main manifestation of the onset of CVT. In patients with unilateral acute hearing impairment, CVT should be included in the diagnosis if the patient had headache or risk factors for venous thrombosis (Gattringer et al., 2012). De-Giorgio et al. (2015) reported a case of CVT that caused sudden death. A CVT patient with superior sagittal sinus involvement that manifested as alexia has also been reported (Thomas et al., 2008).

## Life-style factors

Smoking is a risk factor for cerebrovascular disease, and cerebral infarction is one of the most common causes of death in smokers (Koks et al., 2017). Ciccone et al. (2005) conducted a multicenter, case-control study to explore the relationship between smoking and CVT. To avoid the confounding effects of other risk factors for CVT, a homogeneous subgroup of oral contraceptive users was included. The study enrolled 43 young women with CVT in whom oral contraceptive use was the only known risk factor. A total of 255 healthy oral contraceptive users were included as the control group. The authors compared the prevalence of smoking in the case and control groups and found that it was 26 and 29%, respectively, indicating no significant difference (*p* = 0.7). The authors concluded that in oral contraceptive users, smoking was not associated with CVT. Tufano et al. (2014) conducted a case-control study that compared clinical features and risk factors between 56 CVT patients and 184 age- and sex-matched apparently healthy controls. While they found no significant differences in smoking, overweight (BMI ≥ 25), obesity (BMI ≥ 30) and arterial hypertension between the cases and controls, the rate of oral contraceptive used was significantly different (OR 6.12; *p* < 0.0001). However, smoking-related CVT has also been reported. For example, Raval et al. (Raval and Paul, 2010) reported a case in which CVT was the first manifestation of secondary polycythemia in a smoker. The patient was a 31-year-old male who had suffered a progressive, severe, throbbing headache in the occipital region for 2 weeks. Involuntary motor tics in the left leg were the only signs observed in a neurological examination. MRI and MRA suggested thrombosis of the superior sagittal sinus, right transverse sinus and right sigmoid sinus. Laboratory tests revealed a hemoglobin level of 20 g/dl and a hematocrit of 56.5%. The results of a comprehensive workup for primary polycythemia and hypercoagulable disorders, including an evaluation of JAK2 mutations, were negative. Smoking was the only potential risk factor in this patient. The relationship between smoking and CVT has not been adequately studied, and further studies are therefore required. To explore the relationship between obesity and CVT, Zuurbier et al. (2016a) conducted a case-control study that included 186 cases and 6,134 controls. They found that CVT patients used oral contraceptives more often than did the controls (97 [72.9%] vs. 758 [23.5%] of women). Obesity (BMI ≥ 30) was associated with an increased risk of CVT (adjusted odds ratio [OR], 2.63; 95% CI, 1.53–4.54). Further gender stratification suggested that female obesity was also closely associated with CVT (adjusted OR, 3.50; 95% CI, 2.00–6.14) but that male obesity was not (adjusted OR, 1.16; 95% CI, 0.25–5.30). The authors believed that in the women who use oral contraceptives, overweight and obesity increased the risk of CVT in a dose-dependent manner. In women who did not use oral contraceptives, there was no such association. The relationship between CVT and exercise remains unclear, but case reports have shown that high-intensity physical training may be associated with CVT. For example, Richard et al. (2014) reported an athlete with recurrent CVT who developed thrombosis of the superior sagittal sinus during a marathon training session. The CVT manifested as headache, vomiting and diplopia. No drugs or anabolic steroid consumption were found at the time of onset, and no risk factors were identified after a detailed workup. After 10 months of anticoagulation therapy, the patient's symptoms were completely improved. He again started intensive physical training (10 h of running per week). After 2 months, he was re-admitted to the hospital for progressive headache and cognitive impairment and diagnosed with thrombosis of the straight sinus extending into the right lateral sinus. A more comprehensive examination found no potential risk factors. After 5 months of anticoagulant therapy, the patient recovered again. After discharge, the patient continued to take vitamin K antagonists and performed intensive physical training, even participating in marathons and cycling competitions. After 1 year of follow-up, the CVT had not recurred. More studies are required to explore the relationship between exercise and CVT.

## Treatment

Anticoagulant therapy is the most common treatment for CVT. In the 1990s, three small randomized trials demonstrated the effectiveness of heparin treatment (Einhaupl et al., 1991; Nagaraja et al., 1995; de Bruijn and Stam, 1999). Both low molecular weight heparin (LMWH) and unfractionated heparin (UFH) can be used to treat CVT, but some scholars have proposed that LMWH is more appropriate, except when the patient may need a surgical intervention, in which case, anticoagulation should be immediately reversed (Coutinho et al., 2010; Einhäupl et al., 2010; Misra et al., 2012). Misra et al. (2012) conducted a randomized controlled trial (RCT) in which they compared the efficacy and safety of LMWH and UFH in CVT. Thirty-two patients were administered UFH at a dose of 80 U/kg in an intravenous bolus followed by intravenous infusion at a rate of 18 U/kg/h, and 34 patients were administered LMWH at a dose of 100 units/kg subcutaneously twice daily. Six patient died, all of whom belonged to the UFH group (*P* = 0.01). The authors found that hospital mortality was significantly lower in patients treated with LMWH than in patients treated with UFH. A large multicenter, multi-country study found that patients treated with LMWH had a better functional prognosis after 6 months. Patients treated with LMWH were less likely to develop new intracranial hemorrhage (adjusted odds ratio, 0.29; CI, 0.07–1.3), and this was especially true in those with intracranial lesions at baseline (0.19; CI, 0.04–0.99; Coutinho et al., 2010). In CVT, LMWH and UFH are usually used during the acute phase, and then oral anticoagulants are used later. The best time for anticoagulation therapy remains unclear but is usually administered from 3 to 12 months (Coutinho et al., 2011).

Most patients have a good prognosis after anticoagulation, but some severe CVT patients will exhibit further deterioration of the disease, and intravascular treatment will then be an option. Intravascular thrombolysis treatment includes chemical thrombolysis, mechanical thrombectomy, or both methods at the same time. Li G et al. (Li et al., 2013) treated 52 patients with severe CVT using mechanical thrombectomy combined with injecting 100 to 1,500 × 10^3^ IU of urokinase into the cerebral venous sinus via a guiding catheter at a rate of 4 × 10^3^ IU/min. Of these patients, 87% (45/52) had complete recanalization, 6% (3/52) had partial recanalization, and 7% (4/52) showed no recanalization. Six patients died, and the mRS ranking of the remaining patients were 1.0 ± 0.9, 0.85 ± 0.63, and 0.37 ± 0.53 at discharge, 3-month follow-up and 6-month follow-up, respectively. The authors suggested that mechanical thrombectomy combined with chemical thrombolysis was a safe and effective treatment for severe CVT that did not respond to anticoagulation therapy. Siddiqui et al. (2015) conducted a systematic analysis of 185 CVT patients in 42 studies, all of whom underwent mechanical thrombectomy. Of these patients, 71% underwent chemical thrombolysis at the same time. Sixty percent of the patients had intracranial hemorrhage before surgery, and 47% of the patients were in a coma. The most common complication during the perioperative period was new bleeding (10%). Eighty-four percent of the patients had a good prognosis, and 12% died. It has been proposed that whether intravascular thrombolysis is or is not combined with mechanical bolus, the prognosis is not significantly different (Siddiqui et al., 2014).

Cerebral hernia caused by cerebral edema is the most common cause of death in patients with CVT. In patients with malignant CVT who exhibit continuous and progressive symptoms, decompressive surgery should be performed. Théaudin et al. (2010) reported 12 cases of malignant CVT. Among these patients, all 4 who did not undergo decompressive surgery died (2 cases had unilateral pupil dilatation, and 2 cases had bilateral pupil dilation). There were a total of 3 cases with unilateral pupil dilatation and 2 cases with bilateral pupil dilation in the remaining 8 patents. Among these 8 patients, all underwent decompressive surgery, six had a good prognosis (mRS 0-1), one had a mRS of 3, and one died of pulmonary embolism after surgery. The authors suggested that even in patients with bilateral pupil dilation, decompressive surgery could provide a meaningful benefit. Mahale et al. (2017) conducted a retrospective case analysis of 30 patients with malignant CVT who underwent decompressive craniectomy. More than 2/3 of the patients had a good prognosis, and the authors suggested that patients older than 50 years old, those with a midline shift more than 10 mm, and those in which the basilar cistern had disappeared had a poor prognosis after decompressive craniectomy. Rajan Vivakaran et al. (2012) conducted a study of 34 patients who had undergone decompressive craniectomy. Of these, 76.5% (26/34) had a good prognosis (Glasgow Outcome Scale score of 4 or 5). The authors found that preoperative GCS and immediate postoperative GCS were significantly correlated with poor outcomes.

## Prognosis

Mortality is lower in CVT than in arterial stroke. Over time, mortality in CVT has significantly decreased, and possible explanations include improvements in treatment, a shift in risk factors, and, most importantly, an increase in the number of mild cases that are confirmed using improved diagnostic technologies. The current reported mortality rate is 2–38% (Krayenbuhl, 1968; Wasay et al., 2008; Borhani Haghighi et al., 2012; Nasr et al., 2013).

Most CVT patients have a good prognosis. Approximately 80% of patients have mRS of 0–1, but they usually have residual symptoms and are often unable to return to their previous work. National Institutes of Health Stroke Scale (NIHSS) scores at admission are ≥2, and a low educational level has an impact on both functional recovery and unemployment (Hiltunen et al., 2016). In CVT, a prognosis was not associated with a hypercoagulable state, the number of involved venous sinuses, or intracranial hemorrhage and seizures (Lee et al., 2017).

In CVT, the median time from onset to death is 13 days, and the median time from diagnosis to death is 5 days. The most common cause of death in CVT is cerebral hernia, which is usually caused by multiple intracranial lesions and edema or focal space-occupying lesions. Other causes of death include cardiopulmonary arrest, sudden death, pulmonary embolism, sepsis, and cerebral hypoxia after seizures (Ferro et al., 2001; Canhão et al., 2005). Kowoll et al. (2016) found that in CVT, death was closely related to the mass effect. The authors suggested that the mass effect had an impact on prognosis and that the early detection and treatment of focal space-occupying lesions was a key point in intensive care. The risk factors associated with death in CVT were age >37 years old, male sex, coma, mental status disorder, hemorrhage on admission CT scan, deep CVT thrombosis, infection of the central nervous system, and cancer (Ferro et al., 2004).

## Summary

In general, CVT is a disease with complex clinical manifestations that are atypical in many affected patients. However, in a clinical setting, 73–82.6% of patients exhibit relevant risk factors (Gunes et al., 2016; Palazzo et al., 2017). When patients have an acute onset of a severe headache, progressive aggravation of a chronic intermittent headache, or a new chronic headache with seizures or focal neurological deficits, with or without visual edema, CVT should be considered. In some older patients, headaches may be absent and may only present with paresis or seizures. However, when a patient has isolated cranial nerve paralysis accompanied by persistent symptoms or involving other cranial nerves or exhibits continuously emerging new symptoms, such as intracranial hypertension, paresis, or seizures, CVT should also be included in the diagnosis. If altered consciousness is the main symptom in a CVT patient, the prognosis may be poor. A small number of patients present with atypical symptoms, such as psychiatric symptoms, subarachnoid hemorrhage, and acute hearing impairment. In those patients, attention should be paid to whether the patient has new symptoms, such as headache, hemiplegia, and other manifestations that are common in CVT. When clinical manifestations and risk factors are both taken into consideration, the majority of CVT cases are traceable (Stam, 2005; Uzar et al., 2012). Defining the relationship between lifestyle and CVT will require further research. Most patients with CVT have a good prognosis after anticoagulant therapy, and a minority of patients with malignant CVT may also benefit from endovascular treatment or decompressive surgery. However, most patients are unable to return to their previous work. Because a cerebral hernia is the most common cause of death in CVT, attention should be paid in intensive care to the detection and treatment of focal space-occupying lesions.

## Author contributions

YL: conceived the article and wrote the manuscript. XT and XW: reviewed and edited the manuscript. All authors read and approved the manuscript.

### Conflict of interest statement

The authors declare that the research was conducted in the absence of any commercial or financial relationships that could be construed as a potential conflict of interest.
